# Dyadic Relationships Between Self-Rated Health and Cognition Among Older Adults and Their Spousal Caregivers

**DOI:** 10.1093/geroni/igab046.2004

**Published:** 2021-12-17

**Authors:** Ruotong Liu, Iris Chi, Shinyi Wu

**Affiliations:** 1 University of Southern California, Marina del Rey, California, United States; 2 University of Southern California, University of Southern California, California, United States; 3 University of Southern California, SANTA MONICA, California, United States

## Abstract

This study examines spouses who are in a caregiving situation to discern how they influence each other’s health. Previous studies reported health concordance and cross-domain effects among caregiver and care-recipient dyads. However, it is less understood of the health dyadic relationships among spouses who are in a caregiving situation. No studies have specifically looked into the relationship between self-rated health (SRH) and cognitive functioning among spousal caregiving dyads over time. In this study we analyzed the longitudinal reciprocal relationships between SRH and cognitive functioning measured by the Telephone Interview for Cognitive Status among older adults and their spousal caregivers, and whether the relationship differed by whether husband or wife was the caregiver. Longitudinal data from the Health and Retirement Study (2010-2016) on 540 dyads were pooled and analyzed using structural equation modeling under an actor-partner interdependence model. Results revealed cognitive concordance among older spouses in which caregivers’ cognition is associated with care-recipients’ cognition subsequently (β=0.05, p<.05). SRH concordance was not significant. Cross-domain results showed only one significant direction, that is, care-recipients’ cognition in the subsequent time was significantly correlated with caregivers’ SRH, regardless of whether husband (β=0.09, p<.05) or wife (β=0.08, p<.05) was the caregiver. Our study found that married couples in a spousal caregiving situation displayed cognitive but not overall health concordance, and cross-domain effects of caregiver’s SRH on spousal care recipient’s cognition subsequently. The reciprocal associations suggest that addressing and improving either partner’s physical health and cognition may benefit both dyad members.

